# Long-term surviving influenza infected cells evade CD8^+^ T cell mediated clearance

**DOI:** 10.1371/journal.ppat.1008077

**Published:** 2019-09-26

**Authors:** Jessica K. Fiege, Ian A. Stone, Rebekah E. Dumm, Barbara M. Waring, Brian T. Fife, Judith Agudo, Brian D. Brown, Nicholas S. Heaton, Ryan A. Langlois

**Affiliations:** 1 University of Minnesota, Department of Microbiology and Immunology and the Center for Immunology, Minneapolis, Minnesota, United States of America; 2 Duke University School of Medicine, Department of Molecular Genetics and Microbiology, Durham, North Carolina, United States of America; 3 University of Minnesota, Department of Medicine and the Center for Immunology, Minneapolis, Minnesota, United States of America; 4 Icahn School of Medicine at Mount Sinai, Department of Genetics and Genomic Sciences, New York City, New York, United States of America; St. Jude Children's Research Hospital, UNITED STATES

## Abstract

Influenza A virus (IAV) is a seasonal pathogen with the potential to cause devastating pandemics. IAV infects multiple epithelial cell subsets in the respiratory tract, eliciting damage to the lungs. Clearance of IAV is primarily dependent on CD8^+^ T cells, which must balance control of the infection with immunopathology. Using a virus expressing Cre recombinase to permanently label infected cells in a Cre-inducible reporter mouse, we previously discovered infected club cells that survive both lytic virus replication and CD8^+^ T cell-mediated clearance. In this study, we demonstrate that ciliated epithelial cells, type I and type II alveolar cells can also become survivor cells. Survivor cells are stable in the lung long-term and demonstrate enhanced proliferation compared to uninfected cells. When we investigated how survivor cells evade CD8^+^ T cell killing we observed that survivor cells upregulated the inhibitory ligand PD-L1, but survivor cells did not use PD-L1 to evade CD8^+^ T cell killing. Instead our data suggest that survivor cells are not inherently resistant to CD8^+^ T cell killing, but instead no longer present IAV antigen and cannot be detected by CD8^+^ T cells. Finally, we evaluate the failure of CD8^+^ T cells to kill these previously infected cells. This work demonstrates that additional cell types can survive IAV infection and that these cells robustly proliferate and are stable long term. By sparing previously infected cells, the adaptive immune system may be minimizing pathology associated with IAV infection.

## Author summary

Influenza A virus is a seasonal respiratory pathogen that can cause severe lung damage and death. We previously made the discovery that cells infected with influenza virus do not have a death sentence. An infected cell can survive both influenza virus infection and the immune response to eliminate the virus, specifically CD8^+^ T cells which are required for virus clearance. Here, we investigated how an infected cell could survive the CD8^+^ T cell immune response. We used an influenza virus expressing a recombinant protein that permanently labels infected cells in inducible reporter mice. This system allowed us to detect actively infected cells, as well as cells that had survived influenza virus infection and CD8^+^ T cell-mediated killing, called survivor cells. We demonstrate that survivor cells do not actively block CD8^+^ T cell effector function and are not inherently resistant to CD8^+^ T cell-mediated killing. Our data suggest that survivor cells have lost influenza virus antigen and are rendered invisible to virus-specific CD8^+^ T cells. Our research provides important new insight into the mechanism of how survivor cells can be generated. This could be a mechanism by which the host is protecting the lung from greater pathology during influenza virus infection.

## Introduction

Influenza A virus (IAV) is a negative sense segmented RNA virus causing significant morbidity and mortality annually. IAV has a broad tropism in the respiratory tract infecting club cells, ciliated cells, type I and type II alveolar cells among others [1]. IAV drives destruction of infected cells directly through lytic virus replication and indirectly from the innate and adaptive immune response’s efforts to eliminate the infection. However, we and others, have previously shown IAV infected pulmonary epithelial cells can survive acute IAV infection [2–4]. Survivor cells express a robust interferon simulated gene (ISG) signature, facilitating a transient non-specific antiviral environment in the lung, and protecting against secondary viral infection [2, 4]. Despite the extensive damage caused by viral replication and antiviral immune responses, the lung is able to recover through significant remodeling and repair. While the turnover of pulmonary epithelial cells in the steady state is slow, after injury epithelial cells undergo proliferation to prevent vascular leakage and differentiation to restore lung function [5]. In addition, rare pulmonary progenitor cells are also critical for the repair of the upper and lower airway [6–9].

CD8^+^ T cells are critical for the control of acute IAV infections. CD8^+^ T cells use perforin and granzyme as well as FasL and TRAIL to specifically kill IAV infected cells [10–12]. There are several mechanisms by which cells can evade CD8^+^ T cell-mediated control. Cells can downregulate MHC-I, a strategy employed by quiescent stem cells [13], tumor cells, and cells infected with some viruses [14]. The inhibitory ligand, PD-L1, can bind to PD-1 on CD8^+^ T cells and inhibit T cell effector functions. During IAV infection epithelial cells upregulate PD-L1 resulting in decreased IAV titers [15, 16]. CD8^+^ T cells can also control virus infected cells without destroying the cell. After hepatitis B virus infection, CD8^+^ T cells secrete IFN-γ and TNF-α to stop viral replication and cure infected hepatocytes [17, 18]. It is unclear if survivor epithelial cells are inherently resistant to cytolysis, downregulate MHC-I, or if non-cytolytic control of IAV facilitates their survival and evasion from CD8^+^ T cell-mediated elimination.

In this study we demonstrate that in addition to club cells, ciliated epithelial cells, type I and type II alveolar cells can survive IAV infection. Surviving cells undergo enhanced proliferation compared to uninfected cells after IAV clearance and survivor cells are stable in the lungs until at least 99 days post infection. When we investigated how the adaptive immune system fails to kill survivor cells, we discovered that survivor cells are not inherently resistant to CD8^+^ T cell killing, do not employ active mechanisms to dampen the response and demonstrate that they lack IAV antigen. These results suggest that survivor cells rapidly clear the infection to evade killing from the adaptive immune system. This study sheds new light on the biology of cells that survive acute IAV infection.

## Results

### Multiple epithelial cell types survive acute IAV infection

We generated a H1N1 strain (A/Puerto Rico/8/1934) of IAV expressing Cre recombinase (Cre) (IAV_Cre) downstream of the PA gene. IAV_Cre showed similar replication kinetics to the wt PR8 virus (S1A and S1B Fig) and was cleared between 10 and 12 days post infection (dpi) (S1C Fig). We had previously determined that club cells were the primary cell type surviving IAV infection [2]. However, digestion, processing, sorting and lysing of lung epithelial cells for RNA-seq could bias cell types detected. To determine if a different digestion method could result in a greater breadth of lung epithelial cells recovered, we compared two distinct digestion methods for flow cytometry analysis [2, 19]. Transgenic mice containing a Cre-inducible fluorescent reporter protein were infected with IAV_Cre. With a dispase-based lung digestion, 6-fold more epithelial cells and 10-fold more reporter^+^ epithelial cells were recovered from the lung (S1D and S1E Fig). Therefore, we used this digestion method for all experiments analyzing lung epithelial cells by flow cytometry.

**Fig 1:**
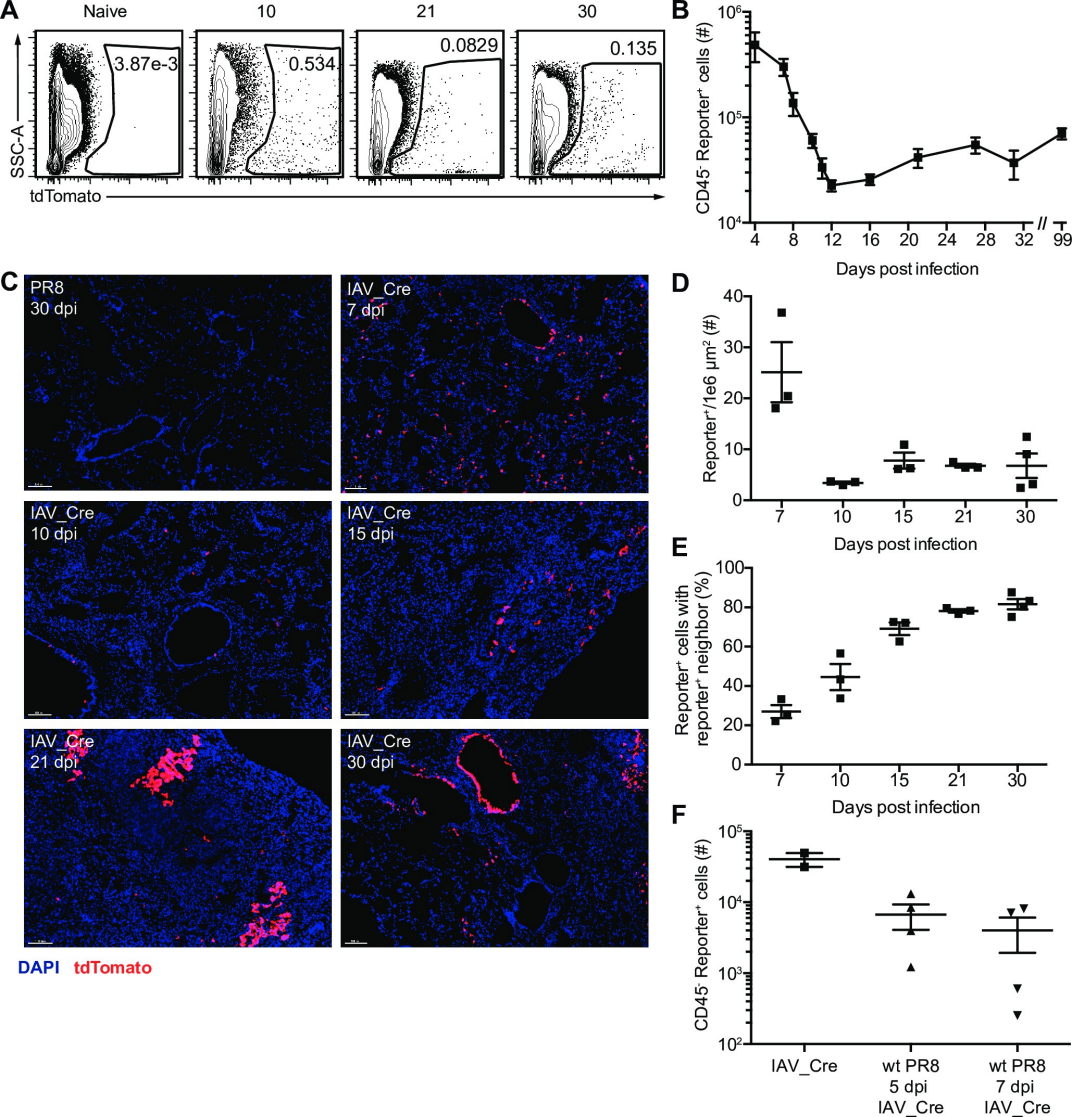
IAV_Cre marks cells lung epithelial cells that survive infection. Cre-inducible reporter mice were infected with wt PR8 or IAV_Cre. (A) Representative flow cytometry plots of CD45^-^ cells from the lungs of IAV_Cre infected mice on indicated dpi. Numbers indicate percentage of tdTomato^+^ cells. Samples were run on different days and gates were set by controls from each individual time point. (B) Number of lung CD45^-^ reporter^+^ cells at indicated dpi. (C) Representative microscopy images of lungs from PR8 or IAV_Cre infected mice on indicated dpi. DAPI (blue) and tdTomato (red). Bars = 100 μm. (D) Number of reporter^+^ cells per 1 x 10^6^ μm^2^. Each point represents a mouse, from two distinct lung sections, which were taken at least 100 μm apart. (E) Percentage of lung reporter^+^ cells with a reporter^+^ neighboring cell. (F) Mice were infected with wt PR8 or IAV_Cre. On 5 or 7 dpi, wt PR8 infected mice were infected with 100,000 PFU of IAV_Cre. Number of lung CD45^-^ reporter^+^ cells at 10 dpi. The results (B) are compiled from multiple independent experiments with at least 4 mice per time point (± SEM). The results (D and E) are compiled from 2 independent experiments with 2–3 mice per group, per experiment (± SEM). The results (F) are from one experiment with at least 2 mice per group (± SEM).

We next determined the presence of reporter^+^ cells by microscopy and flow cytometry. Reporter^+^ lung epithelial cells were observed by flow cytometry at multiple time points after IAV_Cre infection (Fig 1A and S1F Fig). There were large numbers of reporter^+^ epithelial cells early after infection (4–8 dpi), which decreased by 12 dpi, likely to due to lytic IAV replication and CD8^+^ T cell mediated killing (Fig 1B). The number of reporter^+^ epithelial cells remained stable after clearance, and long-term survivor cells could be detected as late as 99 dpi (Fig 1B). We observed similar trends for reporter^+^ cells detected by microscopy (Fig 1C and 1D). Reporter^+^ cells were observed both in the large airways and in the lung parenchyma (Fig 1C). At later time points (15–30 dpi) reporter^+^ cells were frequently observed in clusters with other reporter^+^ cells. The percentage and number of reporter^+^ cells with a reporter^+^ neighbor cell increased from 7 to 30 dpi (Fig 1E and S1G–S1H Fig). As the expression of the fluorescent reporter is a permanent heritable change in the DNA, a reporter^+^ cell with a reporter^+^ neighbor may be the result of a parental infected cell undergoing division.

Surviving cells could have been infected during the first wave of replication or later after induction of antiviral immune responses, or a combination thereof. To determine if survivor cells could be generated late during IAV infection we infected Cre-inducible reporter mice with wt PR8, then infected with IAV_Cre at 5 or 7 dpi and quantified reporter^+^ epithelial cells on 10 dpi. We observed detectable levels of reporter^+^ cells when IAV_Cre was delivered at 5 or 7 dpi (Fig 1F), demonstrating that survivor cells can be generated late during the acute phase of IAV infection, after the induction of antiviral immune responses.

**Fig 2:**
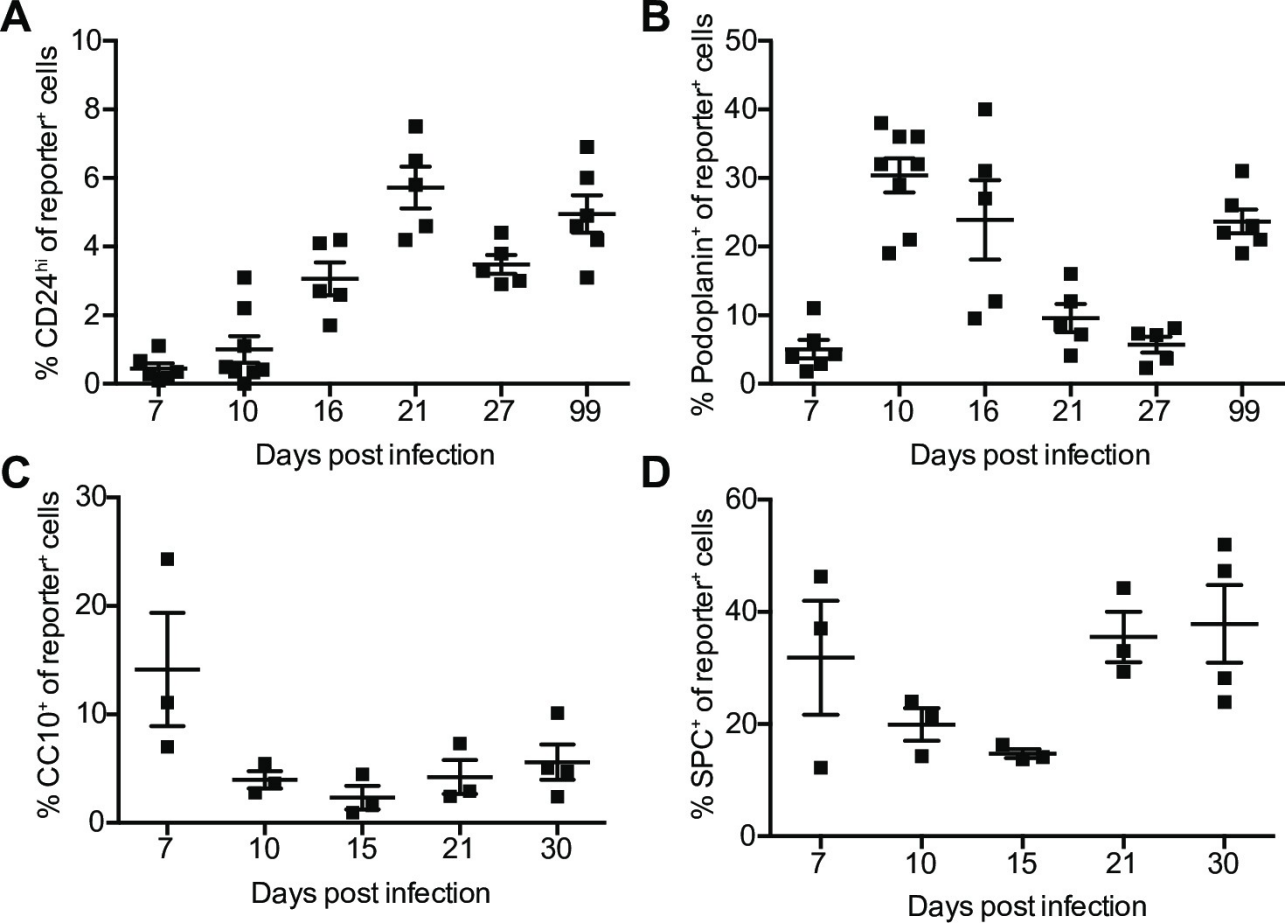
Multiple lung epithelial cell lineages can survive IAV_Cre infection. Cre-inducible reporter mice were infected with IAV_Cre. (A and B) Percentage of lung CD45^-^ reporter^+^ cells that are CD24^hi^ (A), or podoplanin^+^ (B). (C and D) Each point represents a mouse, from two distinct lung sections, which were taken at least 100 μm apart. Percentage of reporter^+^ cells expressing CC10 (C) or SPC (D). The results (A-B) are compiled from multiple independent experiments with at least 4 mice per time point (± SEM). The results (C-D) are compiled from 2 independent experiments with 2–4 mice per group, per experiment (± SEM).

We next assessed if other cell types are able to survive IAV infection and evade CD8^+^ T cell mediated killing, or if survival is a property specific to club cells, as suggested by our previous study [2]. We stained for CD24 (ciliated cells), and podoplanin (type I alveolar cells), to assess cells by flow cytometry (S2 Fig). Total ciliated cells and type I alveolar cells are relatively stable after IAV infection (S2B,E). We observed reporter^+^ cells expressing CD24 or podoplanin at multiple timepoints (Fig 2A and 2B, S2C and S2F Fig). We assessed two other cell types by fluorescence microscopy, club cells (CC10^+^) and type II alveolar cells (SPC^+^) (S3 Fig). We observed reporter^+^ cells expressing CC10 or SPC after clearance (Fig 2C and 2D and S3E and S3F Fig). Together these data demonstrate that in addition to club cells diverse pulmonary epithelial cells can survive acute IAV infection and CD8^+^ T cell-mediated killing.

### Survivor cells proliferate after IAV clearance

**Fig 3:**
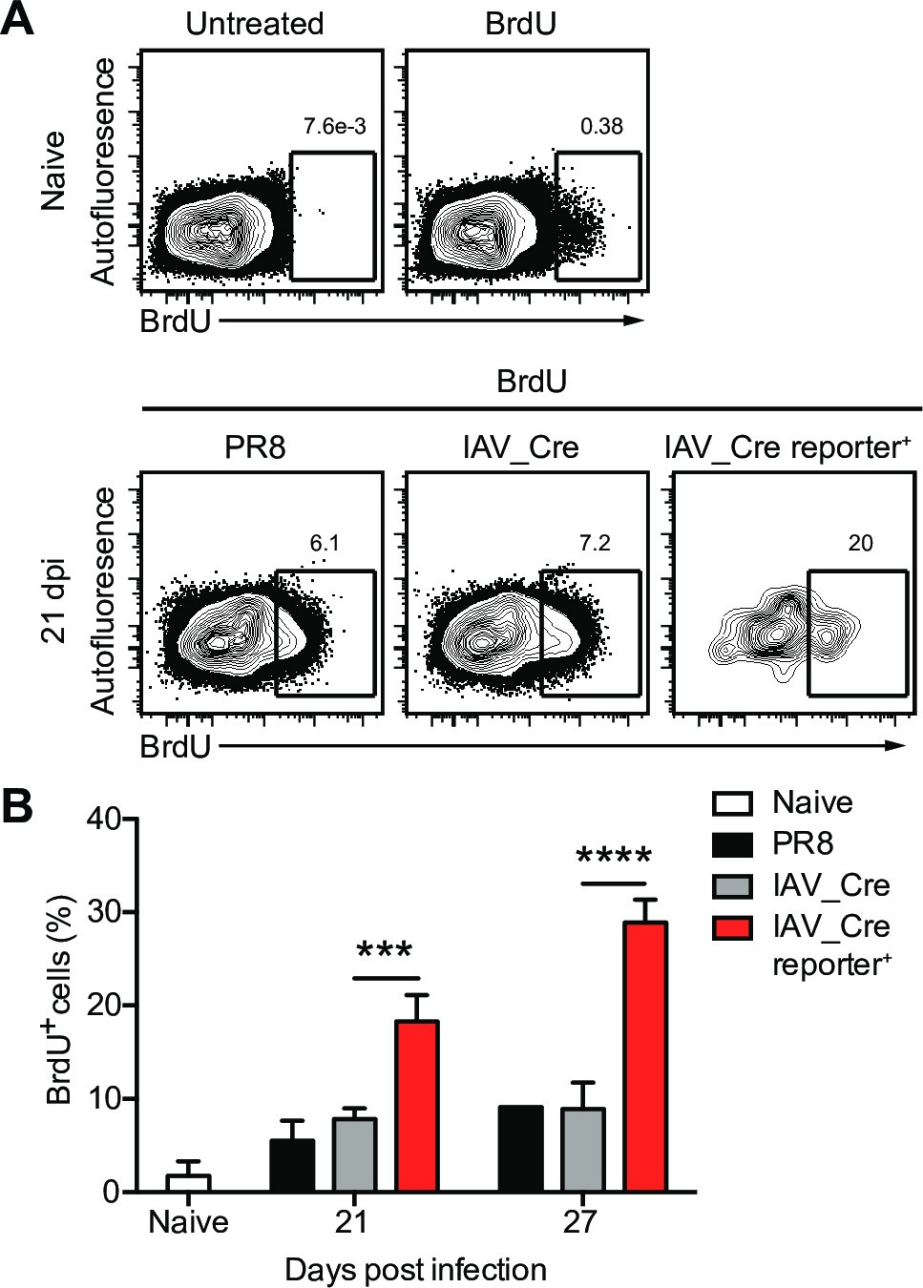
Survivor cells proliferate after IAV clearance. Cre-inducible reporter mice were infected with IAV_Cre. Mice were treated with BrdU i.p. and in the drinking water 6 days prior to euthanasia. (A) Representative BrdU staining of naïve (top) or 21 dpi mice (bottom) untreated or treated with BrdU, gated on CD45^-^ (PR8 and IAV_Cre) or CD45^-^ reporter^+^ (IAV_Cre reporter^+^) lung cells. Numbers indicate percentage of BrdU^+^ cells. (B) Percentage of CD45^-^ (naïve, PR8 or IAV_Cre) or CD45^-^ reporter^+^ (IAV_Cre reporter^+^) cells that are BrdU^+^ at indicated dpi. The results (B) are representative of 2 independent experiments with at least 3 mice per group. Statistically significant differences (unpaired t test) between groups are represented by lines above the bars. *** *p* < 0.001, **** *p* < 0.0001.

We observed that the number of survivor cells increased with time (Fig 1B), and are present in clusters after viral clearance (Fig 1E), indicative of cell division. To assess if the survivor cells are proliferating, we treated mice with BrdU for 6 days prior to euthanasia. We observed a low level of BrdU incorporation in naïve lung epithelial cells (Fig 3A and 3B and S4A and S4B Fig). After IAV clearance the level of BrdU incorporation increased in both wt PR8 and IAV_Cre infected mice when compared to naïve epithelial cells (Fig 3B). Interestingly, a greater percentage of survivor cells had incorporated BrdU than total lung epithelial cells at both 21 and 27 dpi. These data demonstrate that survivor cells are proliferating after IAV clearance, and are doing so at a higher rate than bystander cells after clearance. These data may explain the long-term stability of the surviving cell populations.

### Cellular immune responses do not impact the types of surviving cells

**Fig 4:**
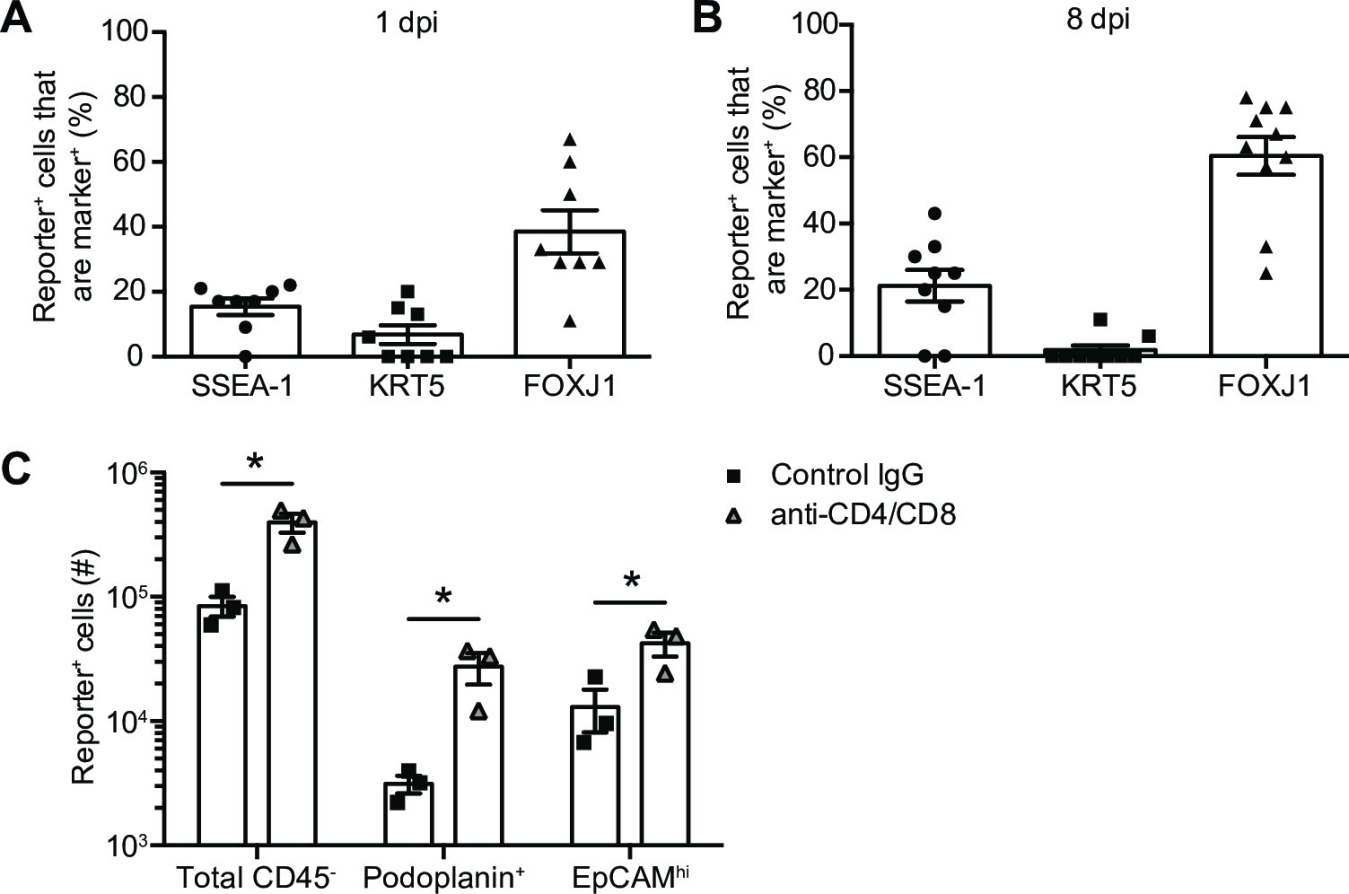
Cellular immune responses do not impact the types of surviving cells. (A-B) Primary differentiated airway epithelial cells from Cre-inducible reporter mice were infected with IAV_Cre at an MOI of 5. Cells were stained with the indicated antibodies and cells stained with cell specific markers were quantified out of total reporter^+^ cells in each image. (A) Percentage of cell marker^+^ cells out of total reporter^+^ cells at 1 dpi. (B) Percentage of cell marker^+^ cells out of total reporter^+^ cells at 8 dpi. (C) Cre-inducible reporter mice were infected with IAV_Cre. On 2, 5 and 8 dpi mice were treated with CD4 and CD8 depletion antibodies or control IgG. Lungs were harvested on 10 dpi and number of lung total CD45^-^ reporter^+^, podoplanin^+^ reporter^+^ and EpCAM^hi^ reporter^+^ cells were enumerated. The results (A and B) are compiled from 2 independent experiments with at least 4 images quantified per group for each experiment. The results (C) are from one experiment with 3 mice per group (± SEM). Statistically significant differences (unpaired t test) between groups are represented by lines above the bars. * *p* < 0.05.

To determine if the immune system exerts bias in the cell subsets that survive IAV infection, we assessed which cells are infected and survive in a primary mouse epithelial cell culture devoid of innate and adaptive immune cells. Tracheal epithelial cells from reporter mice were differentiated at air liquid interface, and infected with IAV_Cre and collected at 1 and 8 dpi, to quantity infected and survivor cell, respectively. In this system, IAV is cleared by 8 dpi (S5D Fig). Using confocal microscopy we demonstrated that the cell types that were initially infected went on the become survivor cells at similar ratios (S5A–S5C Fig, Fig 4A and 4B). These data suggest that in the absence of immune cell killing, a broad array of cell types can survive acute lytic replication.

To determine if these results were consistent in an *in vivo* model, we assessed reporter^+^ cells in mice treated with CD4 and CD8α depletion antibodies or control antibody. These data demonstrated that while the CD8^+^ T cell depletion increased the overall number of reporter^+^ cells there were no major alterations in surviving cell populations (Fig 4C and S5E Fig). Together these data suggest that CD8^+^ T cells do not fail to survey and kill specific cell types.

### Survivor cells do not evade CD8^+^ T cell killing via PD-L1:PD-1 interactions

**Fig 5:**
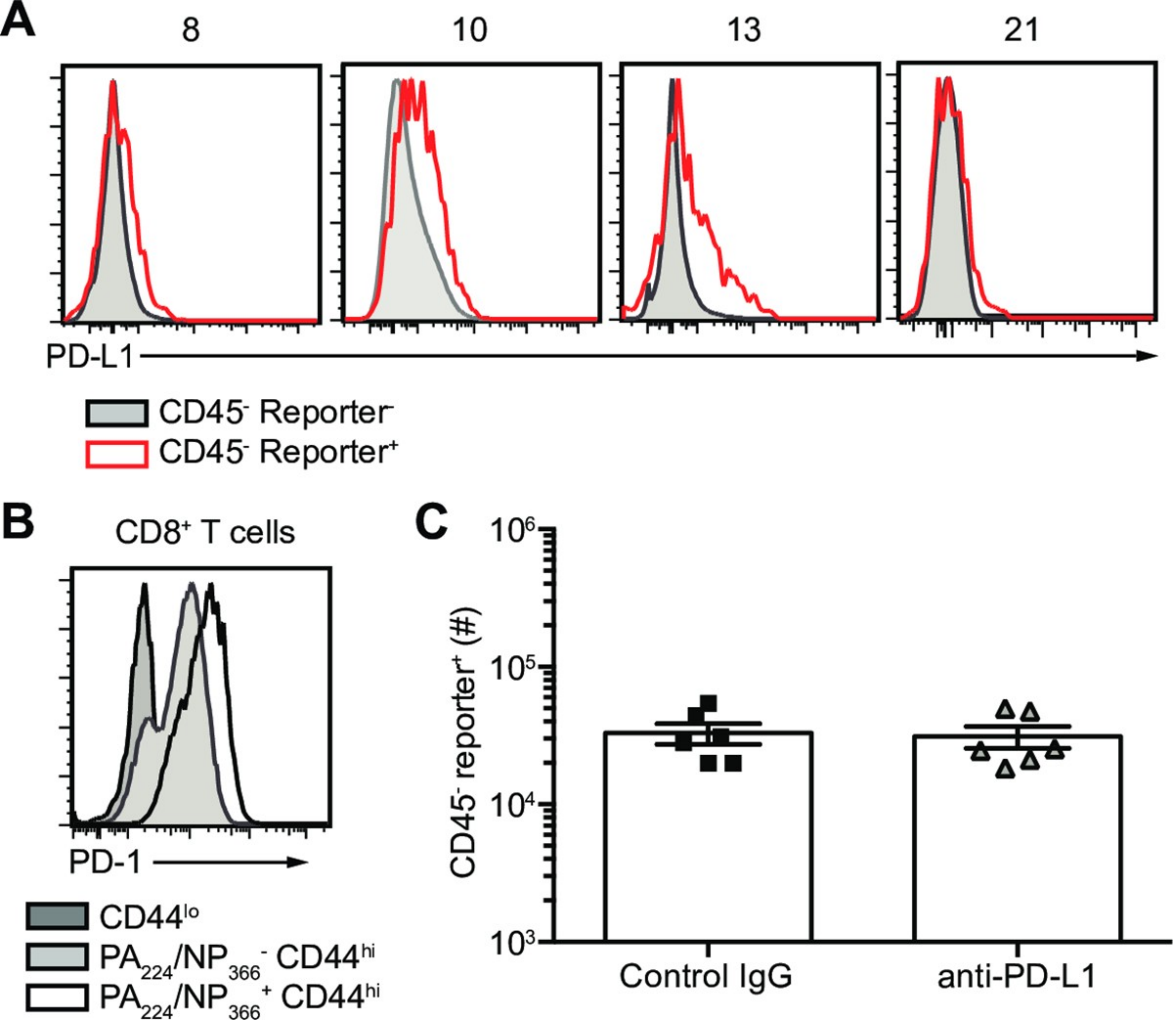
Survivor cells do not use PD-L1 to evade CD8^+^ T cell killing. Cre-inducible reporter mice were infected with IAV_Cre. (A) Representative flow plots of CD45^-^ reporter^+^ (red line) and reporter^-^ (grey shaded) cells assessed for PD-L1 surface expression on indicated dpi. (B) Representative flow plots of PD-1 surface expression on naïve CD44^lo^ CD8^+^ T cells (dark grey shaded), PA_224_/NP_366_^-^ CD44^hi^ CD8^+^ T cells (light grey shaded), and PA_224_/NP_366_^+^ CD44^hi^ CD8^+^ T cells in the lungs of IAV_Cre infected mice on 10 dpi. (C) On 2, 5 and 8 dpi mice were treated with PD-L1 blocking antibody or control IgG. Number of CD45^-^ reporter^+^ cells at 10 dpi. The results (C) are compiled from two independent experiments with 3 mice per group in each experiment (± SEM).

To understand how survivor cells circumvent destruction by IAV-specific CD8^+^ T cells, we assessed our RNA-seq data for molecules known to protect cells from T cell killing [2]. We observed an upregulation of *CD247* (PD-L1) mRNA in reporter^+^ cells at 5 dpi (S6A Fig) [2]. PD-L1 is an inhibitory ligand for PD-1, expressed on CD8^+^ T cells, which suppresses CD8^+^ T cell signaling and killing of target cells [20]. We confirmed increased surface protein expression of PD-L1 on reporter^+^ cells in the lung on 10 and 13 dpi during CD8^+^ T cell-mediated clearance (Fig 5A). Additionally, IAV-specific CD8^+^ T cells in the lung expressed high levels of PD-1 (Fig 5B). To determine if survivor cells use PD-L1 to inhibit CD8^+^ T cell-mediated killing, we treated IAV infected mice with PD-L1 blocking antibody. We chose to block PD-L1 instead of PD-1, to avoid potential interactions between PD-1 and PD-L2, which is expressed on cells other than pulmonary epithelial cells [20]. PD-L1 blocking antibody was given on 2, 5 and 8 dpi and assessed survivor cells at 10 dpi (S6B Fig). There was no difference in the number of survivor cells between control treated and anti-PD-L1 treated mice (Fig 5C). Even when we performed a dual blockade of PD-L1 and PD-1, there was no difference in the number of survivor cells (S6C Fig). These results demonstrate that survivor cell evasion of CD8^+^ T cell killing is not mediated by PD-L1-PD-1 interactions and suggest there is another mechanism of escape.

### Survivor cells are not inherently resistant to CD8^+^ T cell killing

**Fig 6:**
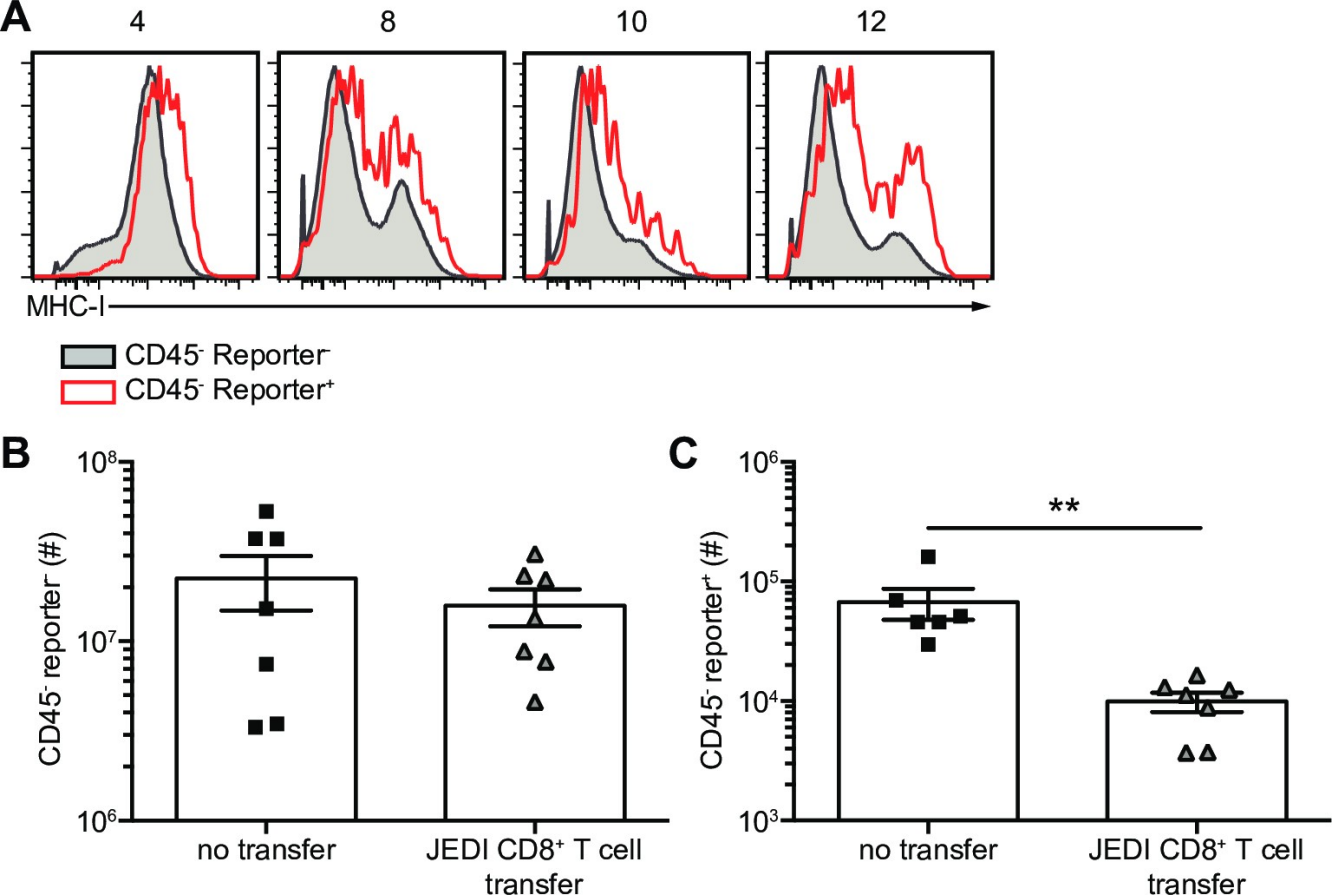
Survivor cells are not inherently resistant to CD8^+^ T cell killing. Cre-inducible reporter mice were infected with IAV_Cre. (A) CD45^-^ reporter^+^ (red line) and reporter^-^ (grey shaded) cells were assessed for MHC-I (H-2K^b^) surface expression. (B and C) JEDI T cells were transferred on 3 dpi and mice were vaccinated with AD_GFP and eGFP_200-208_ peptide. (B) Number of CD45^-^ reporter^-^ cells at 10 dpi. (C) Number of CD45^-^ reporter^+^ cells at 10 dpi. The results (B and C) are compiled from two independent experiments with at least 3 mice per group per experiment (± SEM). Statistically significant differences (unpaired t test) between groups are represented by lines above the bars. ** *p* < 0.01.

Viruses can suppress the surface expression of MHC-I to evade CD8^+^ T cell-mediated clearance. We assessed the mRNA expression of MHC-I (β2M) in lung reporter^-^ and reporter^+^ epithelial cells after IAV_Cre infection. Greater *b2m* mRNA levels were observed on reporter^+^ cells, compared to reporter^-^ epithelial cells (S6D Fig) [2]. MHC-I (H-2K^b^) was also upregulated on the surface of reporter^+^ cells, when compared to total epithelial cells after infection (Fig 6A). It is possible that survivor cells clear IAV infection and no longer display IAV peptide-MHC-I, thereby rendering them invisible from IAV-specific CD8^+^ T cells. It is also possible that survivor cells are inherently resistant to cytolysis.

To assess whether antigen-specific CD8^+^ T cells can kill survivor cells we used a system where IAV_Cre permanently induces a CD8^+^ T cell antigen. To accomplish this, we utilized the just eGFP death-inducing (JEDI) CD8^+^ T cell system, and Cre-inducible eGFP-expressing mice [13, 21, 22]. JEDI T cells recognize eGFP on MHC-I (H-2K^d^), and specifically deplete eGFP expressing cells [13, 21]. To use JEDI T cells in our system we crossed B10.D2 (H-2K^d^) mice to Cre-inducible eGFP expressing mice [21, 22]. F1 mice express H-2K^d^ in all cells and will express eGFP in cells infected with IAV_Cre. Reporter^+^ cells will express eGFP even after IAV is cleared, so we can determine if the survivor cells are resistant to cytolysis or lack viral antigen. H-2K^d^ reporter mice were infected with IAV_Cre, and JEDI T cells were transferred and stimulated with Adenovirus expressing eGFP (AD_eGFP) and eGFP_200-208_ peptide. Survivor cells were then assessed at 10 dpi (S6E Fig). As a control, AD_eGFP was administered to mice and eGFP expression in the lung was restricted to the endothelial (CD31^+^) compartment and was absent from epithelial cells (S6F Fig). Therefore, AD_eGFP priming will not induce CD8^+^ T cell-mediated killing of pulmonary epithelial cells. While the addition of JEDI T cells did not alter the number of reporter^-^ cells in the lung there was a significant decrease in reporter^+^ cells (Fig 6B and 6C). These data demonstrate that survivor cells are susceptible to killing by antigen-specific CD8^+^ T cells, and that survivor cells are not inherently resistant to cytolysis.

We previously demonstrated that survivor cells do not have infectious virus by 10 dpi [2]. However, antigen can have a long half-life and these cells may still present IAV peptide. To determine if survivor cells lack IAV-antigen, we performed an *in vitro* proliferation assay. We generated a new reporter virus containing the GP33 epitope from lymphocytic choriomeningitis virus glycoprotein. The new reporter virus infects epithelial cells to a similar level as the IAV_Cre, and induces a robust GP33-specific CD8^+^ T cell response (S6G and S6H Fig). By incubating *in vitro* activated GP33-specific P14 TCR-Tg CD8^+^ T cells with sorted lung CD45^-^ CD31^-^ reporter^+^ cells from IAV_Cre/NA_GP33 infected mice, we can directly assess if the epithelial cells are presenting antigen. P14 CD8^+^ T cells mixed with 4 dpi reporter^+^ cells undergo division, indicating these epithelial cells express IAV antigen (S6I Fig). In contrast, 10 dpi reporter^+^ cells fail to induce P14 CD8^+^ T cell division (S6I Fig). These data suggest that loss of IAV-antigen may account for the ability of survivor cells to avoid detection and killing by IAV-specific CD8^+^ T cells.

## Discussion

In this study, we sought to elucidate how epithelial cells that survive the lytic phase of IAV infection are able to avoid CD8^+^ T cell mediated killing. We observed that multiple epithelial cell types could survive IAV infection, suggesting that survival is not a cell specific mechanism, and that CD8^+^ T cells are able to survey all areas of the lung. Survivor cells upregulate PD-L1, but do not use PD-L1 to suppress CD8^+^ T cell killing. Survivor cells are not inherently resistant to CD8^+^ T cell killing, do upregulate MHC-I, and no longer express IAV antigen by the time they are under CD8^+^ T cell surveillance.

In our original study, we identified club cells as the primary lung epithelial cell type that survived IAV infection. By incorporating fluorescent microscopy and using an alternative lung digestion method for flow cytometry analysis, we determined that multiple distinct cell types could survive IAV infection. We observed that in addition to club cells, ciliated cells, type I and type II alveolar cells can become survivor cells. We found increased numbers of ciliated epithelial cells and type I alveolar cells long-term compared to acute infection. However, it is difficult to determine if increased presence of these cell types represents a bias in the cells that can survive, increased proliferation, or preferential differentiation into these cell types. In a similar system, Schwemmle and colleagues also observed type I and type II survivor cells [3]. These cells are widespread throughout the lung, requiring CD8^+^ T cells to survey not only a broad array of cells types, but anatomical locations.

The indelible Cre-inducible reporter system allowed us to follow survivor cells out to 99 dpi, demonstrating the longevity and stability of survivor cells. This may be partially due to the slow turnover of many lung epithelial cells [5] and/or capacity to proliferate. As transient antiviral immunity is mediated by surviving club cells [2, 4], we propose that this immunity is lost as survivor cells proliferate and daughter cells lose the ISG expression profile that was acquired during infection. IFN signaling can induce epigenetic changes [23], but it is unclear if these epigenetic changes are heritable in lung epithelial cells.

One interesting finding of our study is increased proliferation of survivor cells compared to bystander cells within the same lung at multiple different time points. Although the benefit of this proliferation is unclear, one potential advantage of increased survivor cell division is that it could aid in the repopulation of the respiratory epithelium. Proliferation of phenotypically altered survivor cells could also alter the pulmonary cytokine and chemokine environment and modulate susceptibility to secondary viral infection [4]. Survivor cells may also proliferate in order to enhance the repair of DNA damage that occurs during viral infection. Some DNA damage repair pathways only function during cell proliferation and DNA repair has been recently demonstrated to help aid in infected cell survival [24]. IAV uses several distinct mechanisms to disrupt cell cycle progression and cell division has been shown to decrease IAV replication [25–27]. Survivor cells could initiate cell division as an antiviral mechanism to blunt virus replication and to dilute antigen to prevent killing by the adaptive immune system. Interestingly, we have previously found that cells infected early have increased activation of genes involved in cell cycle [19].

The cellular events that occur for a survivor cell to be generated are still unclear. Survivor cells can be generated early during infection as well as later during the acute phase after type I and III IFN responses have been generated. As small amounts of Cre recombinase are needed to express the fluorescent reporter, infected cells undergoing abortive replication may turn on the reporter. However, these cells are still impacted by IAV replication as demonstrated by altered ISG profiles [2, 4]. These cells could still express IAV antigen and be targeted by CD8^+^ T cells.

The efforts of the immune system to control IAV drive significant pathology within the lung [28, 29]. Eliminating the virus without killing infected cells could be one way of ameliorating this affect. CD8^+^ T cells have been shown to control hepatitis B virus, sindbis virus, lymphocytic choriomeningitis virus, and simian immunodeficiency virus in the absence of cell lysis [17, 18, 30–33]. Another method to curb pathogenic T cell responses in the lungs is though engagement of inhibitory ligands. Airway epithelial cells increased the expression of PD-L1 after IAV infection [15, 16]. PD-L1 blockade enhanced CD8^+^ T cell-mediated clearance of infected cells [16], increased CD8^+^ T cell numbers and reduced IAV titers [15]. Similar results were observed in PD-1 KO mice during acute IAV infection [34]. Therefore, we hypothesized that this axis may be responsible for protecting surviving cells. However, PD-L1 blockade or systemic blockade of PD-L1 and PD-1 did not result in increased frequency or number of surviving cells. This could be a result of redundancy in inhibitory ligand mediated suppression as others have demonstrated [35, 36]. While inhibitory ligands clearly play a role in IAV clearance and pathogenesis they do not appear to be involved in promoting survival of previously infected cells.

Our data demonstrate that survivor cells are not inherently resistant to CD8^+^ T cell killing. We propose that survivor cells no longer express IAV antigen, thereby rendering the survivor cell invisible to an antigen-specific CD8^+^ T cell. This is consistent with our *in vitro* proliferation assay where only reporter^+^ cells from 4, and not 10, dpi could activate IAV antigen-specific CD8^+^ T cells. There are several mechanisms that could drive this. Cell division could dilute the amount of antigen between two daughter cells, survivor cells could have cleared IAV protein/antigen, and/or survivor cells could have had an abortive infection. Interestingly, while in the presence of JEDI T cells and constitutive antigen there was a substantial decrease in the number of survivor cells, but not all were eliminated. These remaining cells could evade CD8^+^ T cell cytolysis through loss of antigen processing and presentation pathways and/or multiple redundant inhibitory mechanisms.

Together the data presented herein demonstrate that a broad array of epithelial cells are capable of surviving acute lytic virus replication. As these cells are not resistant to killing, these epithelial cells must rapidly clear the infection to evade CD8^+^ T cell mediated cytolysis. This antiviral strategy may be critical for preventing immunopathology.

## Materials and methods

### Mice

C57Bl/6J, B6.Cg-*Gt(ROSA)26Sor*^*tm14(CAG-tdTomato)Hze*^/J, B6.129(Cg)-*Gt(ROSA)26Sor*^*tm4(ACTB-tdTomato*,*-EGFP)Luo*^/J[22], and B10.D2-*Hc*^*0*^ *H2*^*d*^ *H2*-*T18*^*c*^/oSnJ (B10.D2) mice were purchased from The Jackson Laboratory. JEDI mice were obtained from Brian Brown (Mount Sinai). CD45.1 P14 TCR-Tg mice were provided by Dr. Masopust (University of Minnesota).

### IAV infection of mice, BrdU Treatment and antibody depletion and blockade

For IAV infection, mice were anesthetized using a weight-based dose of ketamine/xylazine delivered intraperitoneally (i.p.). Mice were infected intranasally with 40 plaque forming units (PFUs) of PR8, 100 PFU of IAV_Cre, 500 PFU of IAV_Cre/NA_GP33 or 40 PFU IAV_NA_GP33. For sequential infections, mice were infected with 40 PFU of PR8 or 100 PFU of IAV_Cre. PR8 infected mice were infected on 5 or 7 dpi with 100,000 PFU of IAV_Cre. During infection, all mice having weight loss exceeding 25% of their starting weight were sacrificed. For cell proliferation experiments, mice were injected i.p. with 1 mg BrdU (Sigma) once, and given BrdU in drinking water at 0.8 mg/mL for 6 days prior to euthanasia. At indicated dpi lungs were harvested and lung cells were processed and surface staining was performed. Cells were fixed and permeabilized with the BD Cyotfix/Cytoperm kit as per the manufacturer’s instructions, incubated with 1 mg/mL DNase I (Sigma-Aldrich) for 60 min at 37°C washed and stained with anti-BrdU (clone MoBU-1) (Life Technologies). For CD4 and CD8 depletion experiments, IAV infected mice were treated on 2, 5, and 8 dpi with 100 μg of control IgG (SF128) or 100 μg of anti-CD4 (GK1.1) and 100 μg of anti-CD8 (2.43). For PD-L1 blockade experiments, IAV infected mice were treated on 2, 5 and 8 dpi with 250 μg of control IgG (SF128), anti-PD-L1 (M1H6) antibody, or anti-PD-L1 and PD-1 (J43) delivered i.p. [37].

### Flow cytometry and reagents

Single cell suspensions were washed once with 1 X PBS and stained with a fixable viability dye for 30 min on ice, Ghost Dye^™^ Red 780 or UV 450 (Tonbo Biosciences). Cells were washed one time with FACS buffer (ice-cold HBSS supplemented with 2% bovine serum), stained with surface marker Abs, then washed twice before multiparameter flow cytometric detection on a BD LSRFortessa (Becton Dickinson, San Jose, CA). H-2D^b^-PA_224_ (SSLENFRAYV) and H-2D^b^-NP_366_ (ASNENMETM) tetramers were obtained from the National Institutes of Health Tetramer Core Facility. H-2D^b^-GP33 tetramer was a gift from Dr. Vezys. Directly conjugated fluorescent Abs used include: CD24 (clone M1/69), CD44 (clone IM7), CD45 (alone 30-F11), CD45.1 (clone A20), CD45.2 (clone 104), and CD279 (PD-1) (clone 29F.1A12) (Biolegend); CD31 (390) (BD Bioscience); CD247 (PD-L1/B7-H1) (clone M1H5), EpCAM (clone G8.8) and Podoplanin (eBio8.1.1) (eBioscience); CD8-α (clone 53–6.7) and F4/80 (clone BM8) (Tonbo).

### JEDI Experiments

B10.D2 mice were lethally irradiated (1100 rad) and were injected with 5 x 10^6^ bone marrow cells isolated from JEDI mice. At least 8 weeks later, spleen and lymph nodes (cranial, axillary, brachial, inguinal and mesenteric) were harvested, RBCs were lysed and CD8^+^ T cells were negatively selected using the mouse CD8^+^ T cell isolation kit from Stem Cell Technologies following the manufacturer’s instructions. 1 x 10^6^–5 x 10^6^ JEDI T cells were transferred intravenously (i.v.) into IAV_Cre infected B6.129(Cg)-*Gt(ROSA)26Sor*^*tm4(ACTB-tdTomato*,*-EGFP)Luo*^/J x B10.D2 F1 mice. To activate JEDI CD8^+^ T cells, recipient mice were injected i.v. with 1 x 10^8^–2 x 10^8^ transducing units of Adenovirus encoding enhanced green fluorescent protein (AD_eGFP) with 10 μg of eGFP_200-208_ peptide.

### Isolation of epithelial cells from the lung

Mice were euthanized and lungs were inflated via intratrachial injection with 2 mL dispase (Corning) and 0.5 mL 1% low melt agarose (Lonza) and were incubated under an ice pack for 2 minutes. Lungs were removed, transferred to 1 mL dispase and incubated at room temperature for 45 minutes. Lungs were minced into small pieces, and transferred to DMEM with DNaseI (Sigma-Aldrich) 95 U/L and rocked for 10 minutes at room temperature. Lungs were homogenized in a GentleMACS dissociator, filtered through 70 μm filter mesh to generate a single cell suspension, RBCs were lysed and cells were stained for flow cytometry as described above.

### Isolation of lymphocytes from the lung

Mice were euthanized and lungs were harvested, minced into small pieces and washed twice with harvest buffer (ice-cold RPMI 1640 supplemented with 5% bovine serum, 4 mM L-glutamate and 10 mM HEPES). Lungs were incubated in a solution of RPMI 1640/ 10% bovine serum/ 2 mM MgCl_2_/ 2mM CaCl_2_/ 10 mM HEPES/ 4 mM L-glutamate medium containing 100 U/mL of collagenase type I (Worthington) for 45 min at 37°C. Lung pieces were then incubated in a solution of RPMI 1640/ 10% bovine serum/ 10 mM HEPES/ 4 mM L-glutamate medium containing 1.3 mM EDTA (Calbiochem) for 45 min at 37°C. Single cell suspensions of all tissues were generated using a Gentle MACS dissociator and cells were passed through 70 μm filter mesh, RBC lysed, and stained for flow cytometry as described above.

### *In vitro* proliferation assay

Spleen and lymph nodes (cranial, axillary, brachial, inguinal and mesenteric) were harvested from a CD45.1 P14 TCR-Tg mouse. RBCs were lysed and CD8^+^ T cells were negatively selected using the mouse CD8^+^ T cell isolation kit from Stem Cell Technologies following the manufacturer’s instructions. 1 x 10^6^ P14 T cells were placed in 24 well plates pre-treated with anti-CD3 and anti-CD28 (10 μg/mL of each). P14 T cells were incubated at 37°C for 48 hours in the presence of 0.0025 μg/mL IL-12 (R&D Systems). After 48 hours, cells were washed and plated at 5 x 10^5^ cells/mL in the presence of 10 U/mL recombinant human IL-2 for 24 hours. P14 T cells were labeled with CellTrace Violet (CTV; Invitrogen) per the manufacturer’s instructions. P14s were mixed at a 10:1 ratio with sorted CD45^-^ CD31^-^ reporter^+^ cells from the lungs of B6.129(Cg)-*Gt(ROSA)26Sor*^*tm4(ACTB-tdTomato*,*-EGFP)Luo*^/J mice infected with IAV_Cre/NA_GP33 4 or 10 days prior. After 48 hours, cells were stained for flow cytometry. Cell cultures were maintained in RPMI 1640 supplemented with 10% FCS. 4mM L-glutamine, 0.1 mM non-essential amino acids, 1 mM sodium pyruvate, 100 U/mL penicillin and streptomycin and 10 mM HEPES.

### Plasmid design & virus rescue

In-Fusion primers (Takara Bio, Inc.) were designed for insertion of a PA2A site followed by Cre and the complete 5’ vRNA packaging signal (184 nts) into PA of influenza A/Puerto Rico/8/1934 (PR8)[2, 38]. PA_Cre was then cloned into pDZ rescue system via In-Fusion HD cloning. NA_GP33 was inserted into the stalk of NA at a position amenable to insertion via infusion cloning as described [39, 40]. All viruses were rescued via HEK293T transfection and amplified in embryonated chicken eggs as previously described [41]. Rescued viruses were sequence confirmed and titered on Madin-Darby canine kidney (MDCK) cells (ATCC).

### Cell culture and plaque assay

MDCK cells were maintained in Dulbecco’s modified Eagle medium (DMEM) with 10% fetal bovine serum (FBS) and 1% penicillin-streptomycin (pen-strep). Infections were carried out in infection medium (phosphate buffered saline [PBS] with 10% CaMg, 1% pen-strep, 5% bovine serum albumin [BSA]) at 37°C for 1 hr. Infection medium was replaced with an agar overlay (MEM, 1 mg/mL tosylsulfonyl phenylalanyl chloromethly ketone [TPCK] trypsin, 1% DEAE-dextran, 5% NaCO_3_, 2% agar), and cells were cultured at 37°C for 40 h and then fixed with 4% formaldehyde. Blocking and immunostaining were done for 1 hr at 25°C in 5% milk using the following antibodies: polyclonal anti-IAV PR8/34, 1:5,000 (V301-511-552), and peroxidase rabbit anti-chicken IgG, 1:5,000 (303-035-003; Jackson Immuno Research). TrueBlue peroxidase substrate (50-647-28; Kirkegard & Perry Laboratories) was used as directed for detection of virus plaques.

### Tissue preparation and microscopy

B6.Cg-*Gt(ROSA)26Sor*^*tm14(CAG-tdTomato)Hze*^/J mice previously infected with PR8 or IAV_Cre were euthanized at indicated dpi. Lungs were harvested and fixed in 2% PFA for two hours, immersed overnight in 30% sucrose, inflated and flash frozen in optimum cutting temperature compound (O.C.T.). 7 μm sections were cut from each block with a Leica CM 1860 cryostat and stained prior to imaging on a Leica DM6000B EPI fluorescent microscope (violet LED). Histo-cytometry was performed using Imaris (Bitplane) by creating surfaces for the reporter^+^ cells (tdTomato) and markers (SPC or CC10), and performing a distance transformation to detect double positive cells. Reporter^+^ cells were manually counted in ImageJ. Neighbor cells were defined as two or more reporter^+^ DAPI^+^ cells within the proximity of one DAPI-stained nucleus to one another. Abs used were: CC10 (polyclonal) (Abcam), proSP-C (polyclonal) (Millipore Sigma), goat anti-rabbit (polyclonal) (ThermoFisher Scientific).

### Primary mouse pulmonary epithelial cell cultures

Tracheal epithelial cells were collected from the lungs of Cre-inducible reporter mice and plated on 4 μm pore transwell membranes (Corning). Cultures were grown in mTec^+^ for 2 days of proliferation then switched to mTec-serum free media for differentiation. After 21 days of differentiation, the pseudostratified cultures were infected with IAV_Cre then harvested at 1 dpi (to measure actively infected cells) and 8 dpi (to measure survivor cells after the clearance of viral infection). These cultures were then fixed with 2% PFA and antigen retrieval was performed in a citrate buffer. Samples were then stained with antibodies for active infection (HA, PY102), basal stem cells (Krt5, Biolegend, clone Poly19055, cat 905501), ciliated cells (FoxJ1, eBioscience, clone 2A5, cat 14–9965), secretory cells (SSEA-1, Biolegend, clone MC-480, cat 125611) using tdTomato as an endogenous marker of infection. Wholemount membranes were mounted in Prolong Diamond (Life Technologies) then imaged on a SP5 inverted confocal microscope (Leica) and images processed using Fiji.

### Statistical analysis

GraphPad Prism (version 6.0h, GraphPad Software, La Jolla, CA) was used to determine statistical significance using as Student unpaired two-tailed *t* test. A *p* value of < 0.05 was considered statistically significant.

### Ethics statement

Care and use of the animals was in accordance with The guide for the Care and Use of Laboratory Animals from the National Research Council and the USDA Animal Care Resource Guide. All experimental protocols involving the use of mice were approved by the Institutional Animal Care and Use Committee at the University of Minnesota (protocol: 1708-35040A. approved 09/14/2017; expires 09/13/2020). Eggs were obtained from Charles River Laboratories and were grown at 37°C until 12 days of embryonation.

## Supporting information

S1 Fig IAV_Cre system and lung digestion protocol dictates number of epithelial cells analyzed by flow cytometry. (A-E) Mice were infected with 40 PFU of wt PR8 or 100 PFU of IAV_Cre. (A) Mice were monitored for weight loss. (B) Lung titer was determined on 2 and 5 dpi by plaque assay. (C) Lung titer was determined on 8, 10 and 12 dpi by plaque assay. (D and E) On 11 dpi lungs were harvested and digested with collagenase or dispase. (D) Number of CD45^-^ cells yielded from each protocol. (E) Number of CD45^-^ reporter^+^ cells yielded from each protocol. (F) Representative flow cytometry plots of total lung cells on indicated dpi. (G) Number of reporter^+^ cells with a reporter^+^ neighbor. (H) Number of reporter^+^ cells without a reporter^+^ neighbor. The data (A) is a minimum of 4 mice per time point, combined experiments. The data (B) is from one experiment, with 3 mice per group. The data (C) is from one experiment, with at least 4 mice per group. The results (D and E) are from one experiment with 2 mice per group. The results (H and G) are compiled from 2 independent experiments with 2–3 mice per group, per experiment (± SEM). Statistically significant differences (unpaired t test) between groups are represented by lines above the bars. *p < 0.05, *** *p* < 0.001.
(TIF)

S2 Fig Multiple lung epithelial cell lineages can survive IAV_Cre infection, detected by flow cytometry. Cre-inducible reporter mice were infected with IAV_Cre. (A and D) Representative flow cytometry plots of CD24 (A) or podoplanin (D) expression on epithelial cells from the lungs of naïve or IAV_Cre infected mice on 10 dpi, either CD45^-^ reporter^-^ or CD45^-^ reporter^+^. Numbers indicate percentage of CD24^hi^ (A) or podoplanin^+^ (D) cells. (B and E) Percentage of lung CD45^-^ reporter^-^ cells that are CD24^hi^ (B), or podoplanin^+^ (E). (C and F) Number of lung CD45^-^ reporter^+^ cells that are CD24^hi^ (C) or podoplanin^+^ (F). The results (B-C and E-F) are compiled from multiple independent experiments with at least 4 mice per time point (± SEM).
(TIF)

S3 Fig Multiple lung epithelial cell lineages can survive IAV_Cre infection, detected by fluorescent microscopy. Cre-inducible reporter mice were infected with IAV_Cre. (A-B) Representative microscopy images of lungs on indicated dpi. DAPI (blue), tdTomato (red) and CC10 (green) (A) or SPC (green) (B). Bars = 100 μm. (C-D) Each point represents a mouse, from two distinct lung sections, which were taken at least 100 μm apart. (C and D) Number of CC10^+^ (C) or SPC^+^ (D) cells per 1 x 10^6^ μm^2^. (E and F) Number of CC10^+^ reporter^+^ (E) or SPC^+^ reporter^+^ (F) cells per 1 x 10^6^ μm^2^. The results (C-F) are compiled from 2 independent experiments with 3–4 mice per group, per experiment (± SEM).
(TIF)

S4 Fig Survivor cells proliferate after IAV clearance. Mice were treated as in Fig 3. (A) Representative flow cytometry plots of total lung cells on indicated dpi. (B) Number of total CD45^-^ reporter^+^ cells and BrdU^+^ CD45^-^ reporter^+^ cells at indicated dpi. The results (B) are representative of 2 independent experiments with at least 3 mice per group (± SEM).
(TIF)

S5 Fig Tracheal epithelial cells differentiated in vitro survive IAV replication. (A-C) Representative images of primary differentiated airway epithelial cells derived from Cre-inducible reporter mice. Cultures were infected with IAV_Cre at an MOI of 5 and collected at 1 and 8 dpi to assess infected and survivor cells, respectively. Cells were identified as infected based on tdTomato expression (red) and stained with the indicated antibodies (green). (A) SSEA-1 (secretory cells) (B) KRT5 (basal stem cells) (C) FOXJ1 (ciliated cells). scale bar = 10 μm, yellow arrows = marker^+^ reporter^+^ cells, white arrows = marker^+^ reporter^-^cells. (D) Percentage of primary differentiated airway epithelial cells positive for HA at indicated dpi. (E) Cre-inducible reporter mice were infected with wt PR8 or IAV_Cre and treated with control or anti-CD4/CD8 antibodies as in Fig 4C. Representative flow cytometry plots of lung CD45^-^, CD45^-^ podoplanin^+^, and CD45^-^ EpCAM^+^ cells on 10 dpi. The results (D) are representative of 2 independent experiments with 5 wells per time point (± SEM).
(TIF)

S6 Fig Survivor cells express PD-L1 and MHC-I mRNA. (A and C-D) Cre-inducible reporter mice were infected with IAV_Cre. (A and D) Live, CD45^-^ reporter^+^ and reporter^-^ cells were FACS sorted from IAV_Cre infected mice at indicated dpi and assessed by mRNA-seq.[2] (A) mRNA reads were analyzed for *CD247* (PD-L1) at indicated dpi. (B) Schematic of control Ig or anti-PD-L1 treatment of infected mice in Fig 4. (C) On 2, 5 and 8 dpi mice were treated with PD-1 and PD-L1 blocking antibody or control IgG. Number of CD45^-^ reporter^+^ cells at 10 dpi. (D) mRNA reads were analyzed for *b2m* (β2M) at indicated dpi. (E) Schematic of JEDI T cell transfer and AD_eGFP infection in Fig 5. (F) Representative flow cytometry plots of lung CD45^-^ CD31^-^, CD31^+^ and CD45^+^ cells 7 days after AD_eGFP vaccination. (G-I) Cre-inducible reporter mice were infected with IAV_Cre, IAV_Cre/NA_GP33 or IAV_NA_GP33. (G) Number of lung CD45^-^ reporter^+^ cells at 4 dpi. (H) Number of lung CD8^+^ CD44^+^ H-2D^b^-GP33^+^ T cells at 10 dpi. (I) CD45^-^ CD31^-^ Reporter^+^ cells were sorted from lungs of mice infected 4 or 10 days prior with IAV_Cre/NA_GP33. Sorted cells were mixed with *in vitro* activated P14 CD8^+^ T cells labeled with CTV. After 48 hours cells were analyzed by flow cytometry. Percentage of P14 CD8^+^ T cells that have divided. Data (A and D) were obtained from ref [2]. The results (C) are from 1 experiment with 3 mice per group (± SEM). The results (G) are representative of 3 independent experiments (± SEM). The results (H) are from 1 experiment with 2 mice per group (± SEM). The results (I) are representative of 2 independent experiments (± SEM).
(TIF)
