# Supplementary figures and images for: Long-term surviving influenza infected cells evade CD8+ T cell mediated clearance
Source: PLoS Pathog. 2019 Sep 26;15(9):e1008077. doi: 10.1371/journal.ppat.1008077 (PMC6782110; doi:10.1371/journal.ppat.1008077)

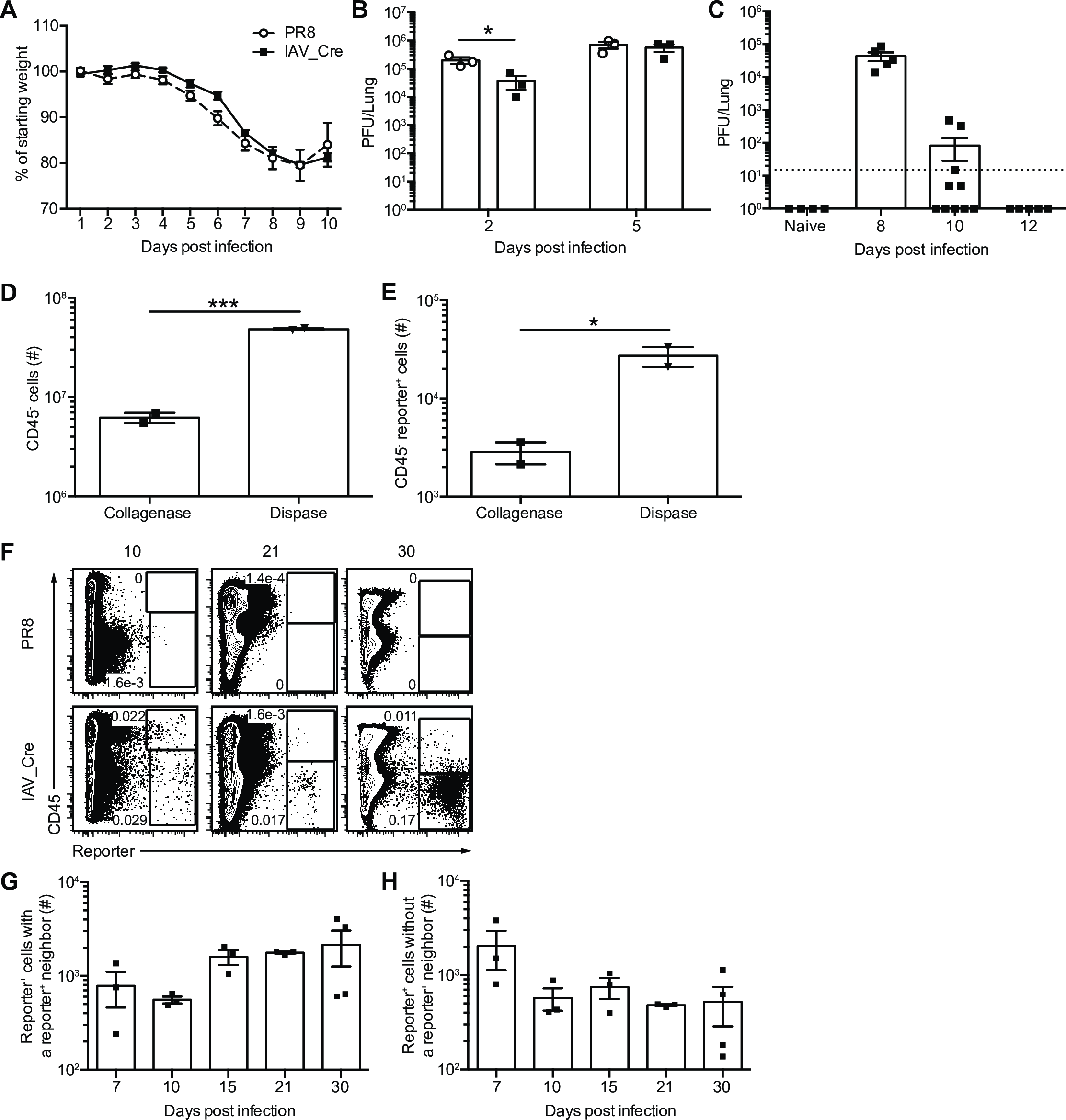

Supplement: S1 Fig — (A-E) Mice were infected with 40 PFU of wt PR8 or 100 PFU of IAV_Cre. (A) Mice were monitored for weight loss. (B) Lung titer was determined on 2 and 5 dpi by plaque assay. (C) Lung titer was determined on 8, 10 and 12 dpi by plaque assay. (D and E) On 11 dpi lungs were harvested and digested with collagenase or dispase. (D) Number of CD45- cells yielded from each protocol. (E) Number of CD45- reporter+ cells yielded from each protocol. (F) Representative flow cytometry plots of total lung cells on indicated dpi. (G) Number of reporter+ cells with a reporter+ neighbor. (H) Number of reporter+ cells without a reporter+ neighbor. The data (A) is a minimum of 4 mice per time point, combined experiments. The data (B) is from one experiment, with 3 mice per group. The data (C) is from one experiment, with at least 4 mice per group. The results (D and E) are from one experiment with 2 mice per group. The results (H and G) are compiled from 2 independent experiments with 2–3 mice per group, per experiment (± SEM). Statistically significant differences (unpaired t test) between groups are represented by lines above the bars. *p < 0.05, *** p < 0.001. (TIF) [file ppat.1008077.s001.tif]

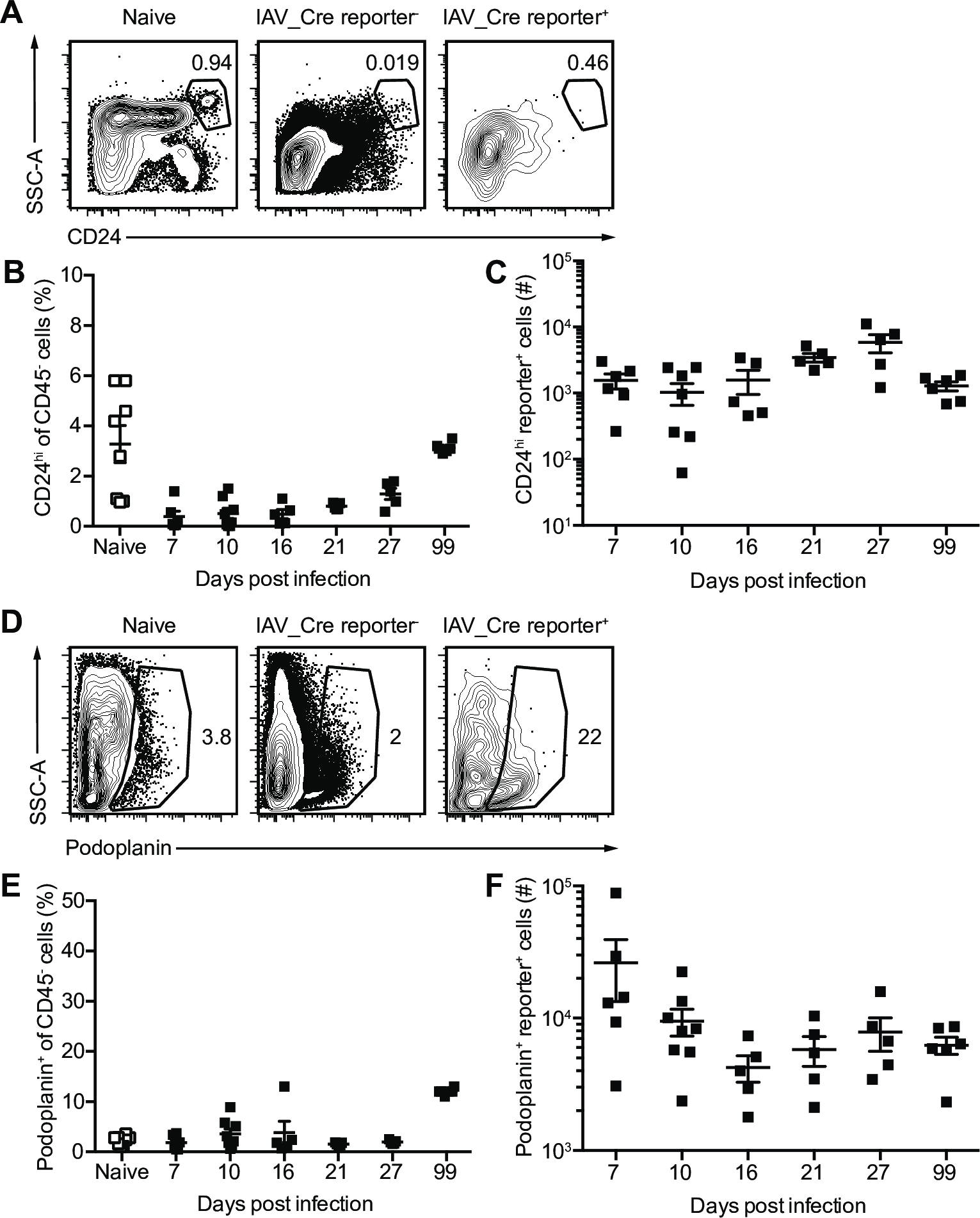

Supplement: S2 Fig — Cre-inducible reporter mice were infected with IAV_Cre. (A and D) Representative flow cytometry plots of CD24 (A) or podoplanin (D) expression on epithelial cells from the lungs of naïve or IAV_Cre infected mice on 10 dpi, either CD45- reporter- or CD45- reporter+. Numbers indicate percentage of CD24hi (A) or podoplanin+ (D) cells. (B and E) Percentage of lung CD45- reporter- cells that are CD24hi (B), or podoplanin+ (E). (C and F) Number of lung CD45- reporter+ cells that are CD24hi (C) or podoplanin+ (F). The results (B-C and E-F) are compiled from multiple independent experiments with at least 4 mice per time point (± SEM). (TIF) [file ppat.1008077.s002.tif]

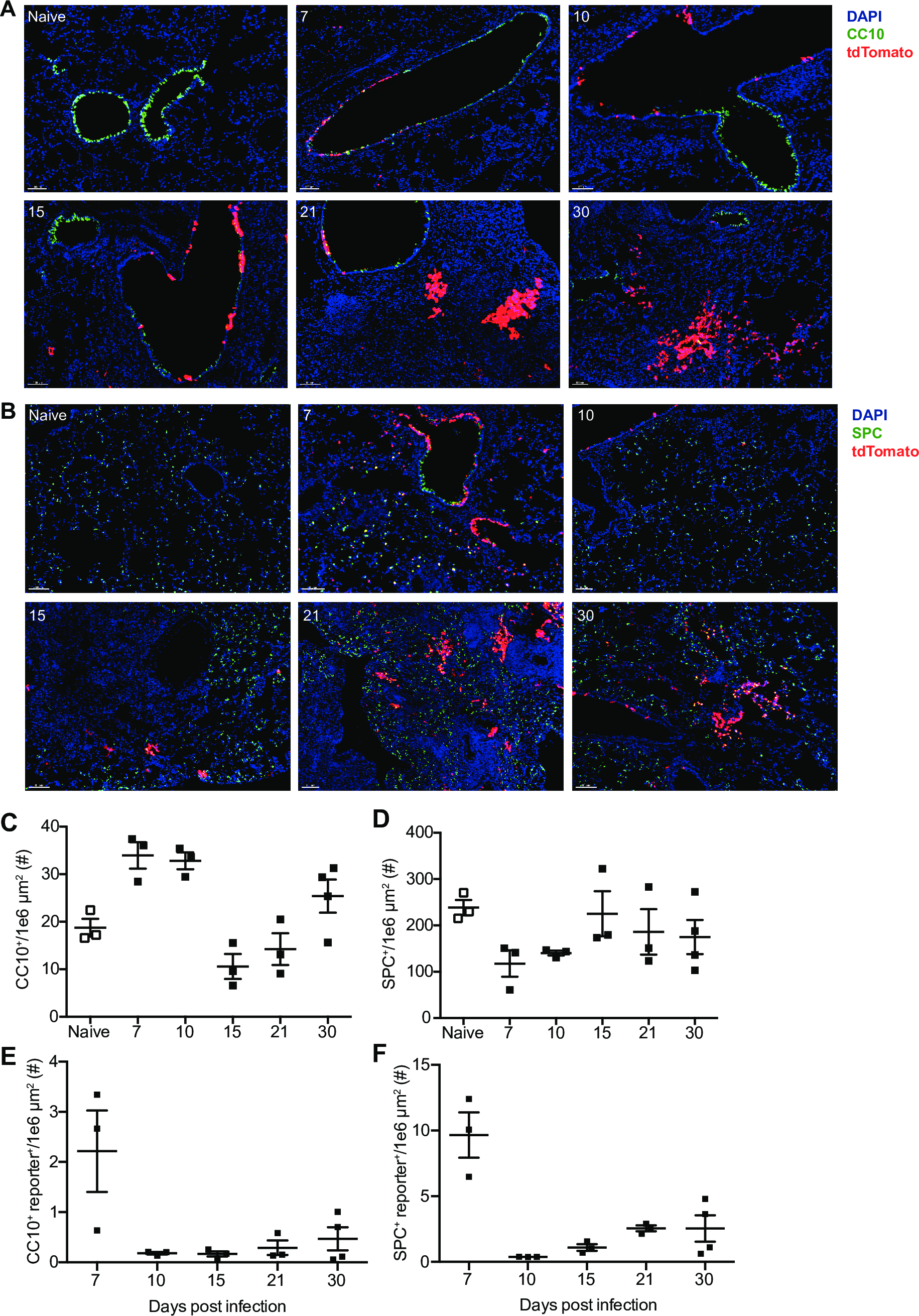

Supplement: S3 Fig — Cre-inducible reporter mice were infected with IAV_Cre. (A-B) Representative microscopy images of lungs on indicated dpi. DAPI (blue), tdTomato (red) and CC10 (green) (A) or SPC (green) (B). Bars = 100 μm. (C-D) Each point represents a mouse, from two distinct lung sections, which were taken at least 100 μm apart. (C and D) Number of CC10+ (C) or SPC+ (D) cells per 1 x 10^6^ μm2. (E and F) Number of CC10+ reporter+ (E) or SPC+ reporter+ (F) cells per 1 x 10^6^ μm2. The results (C-F) are compiled from 2 independent experiments with 3–4 mice per group, per experiment (± SEM). (TIF) [file ppat.1008077.s003.tif]

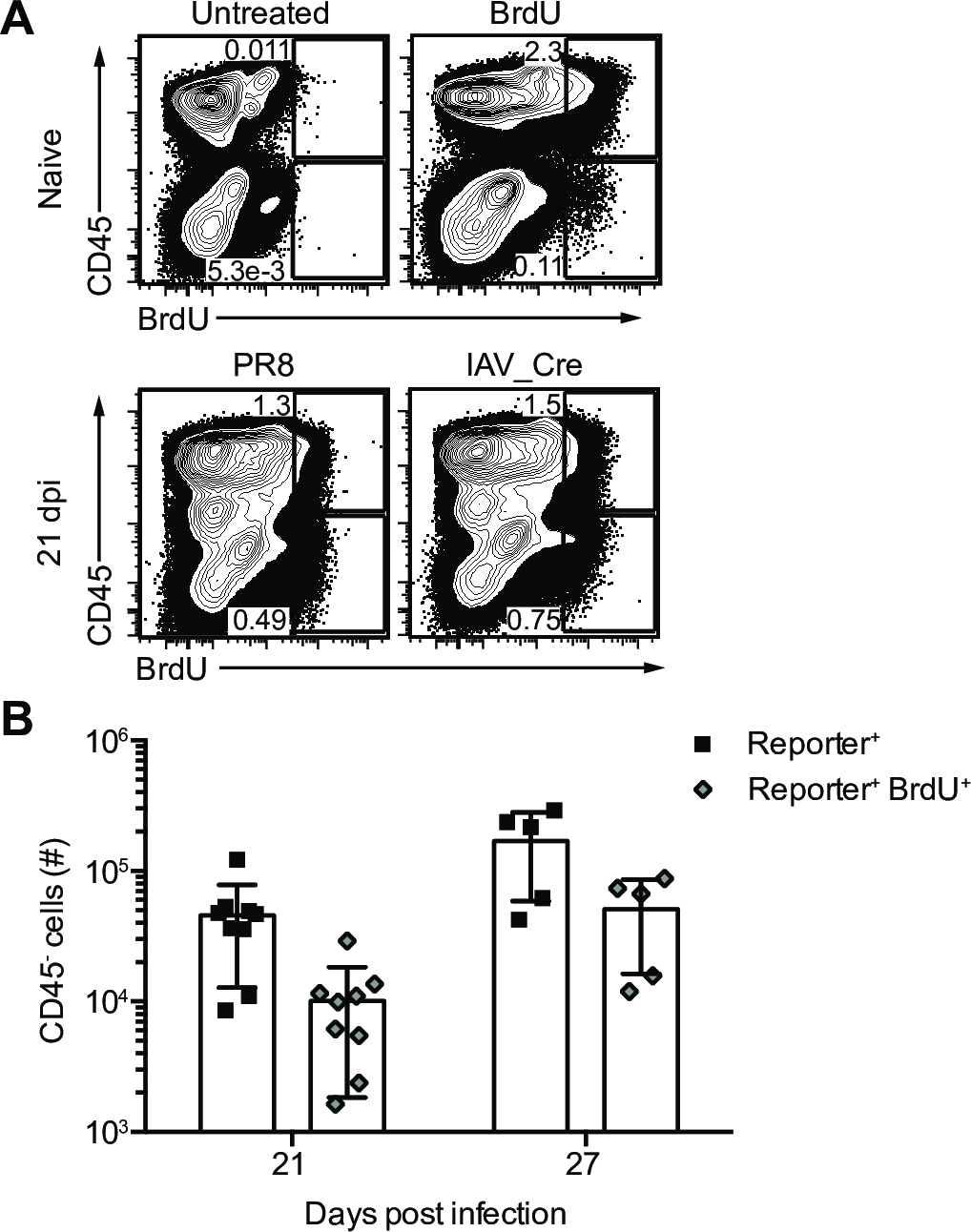

Supplement: S4 Fig — Mice were treated as in Fig 3. (A) Representative flow cytometry plots of total lung cells on indicated dpi. (B) Number of total CD45- reporter+ cells and BrdU+ CD45- reporter+ cells at indicated dpi. The results (B) are representative of 2 independent experiments with at least 3 mice per group (± SEM). (TIF) [file ppat.1008077.s004.tif]

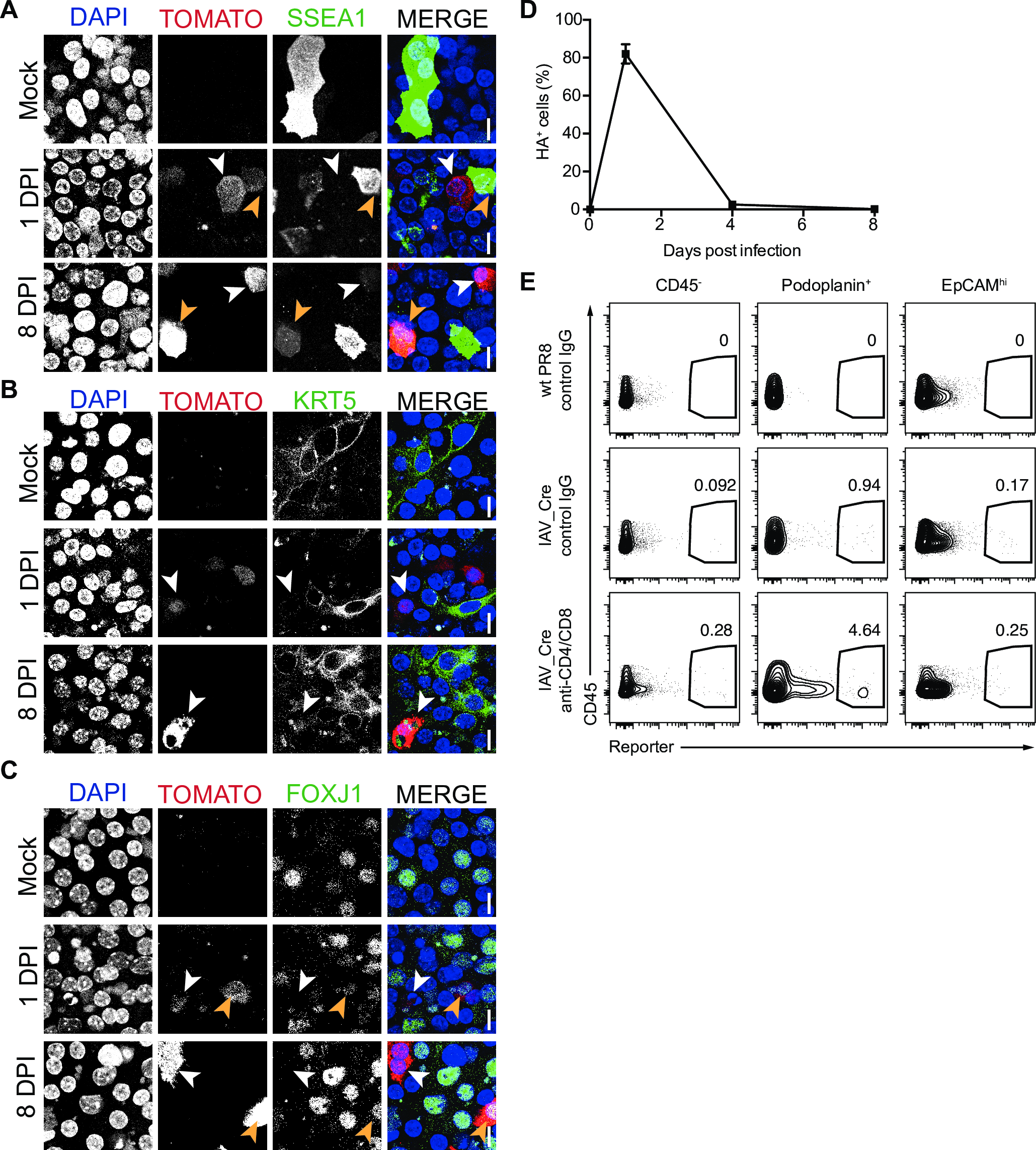

Supplement: S5 Fig — (A-C) Representative images of primary differentiated airway epithelial cells derived from Cre-inducible reporter mice. Cultures were infected with IAV_Cre at an MOI of 5 and collected at 1 and 8 dpi to assess infected and survivor cells, respectively. Cells were identified as infected based on tdTomato expression (red) and stained with the indicated antibodies (green). (A) SSEA-1 (secretory cells) (B) KRT5 (basal stem cells) (C) FOXJ1 (ciliated cells). scale bar = 10 μm, yellow arrows = marker+ reporter+ cells, white arrows = marker+ reporter-cells. (D) Percentage of primary differentiated airway epithelial cells positive for HA at indicated dpi. (E) Cre-inducible reporter mice were infected with wt PR8 or IAV_Cre and treated with control or anti-CD4/CD8 antibodies as in Fig 4C. Representative flow cytometry plots of lung CD45-, CD45- podoplanin+, and CD45- EpCAM+ cells on 10 dpi. The results (D) are representative of 2 independent experiments with 5 wells per time point (± SEM). (TIF) [file ppat.1008077.s005.tif]

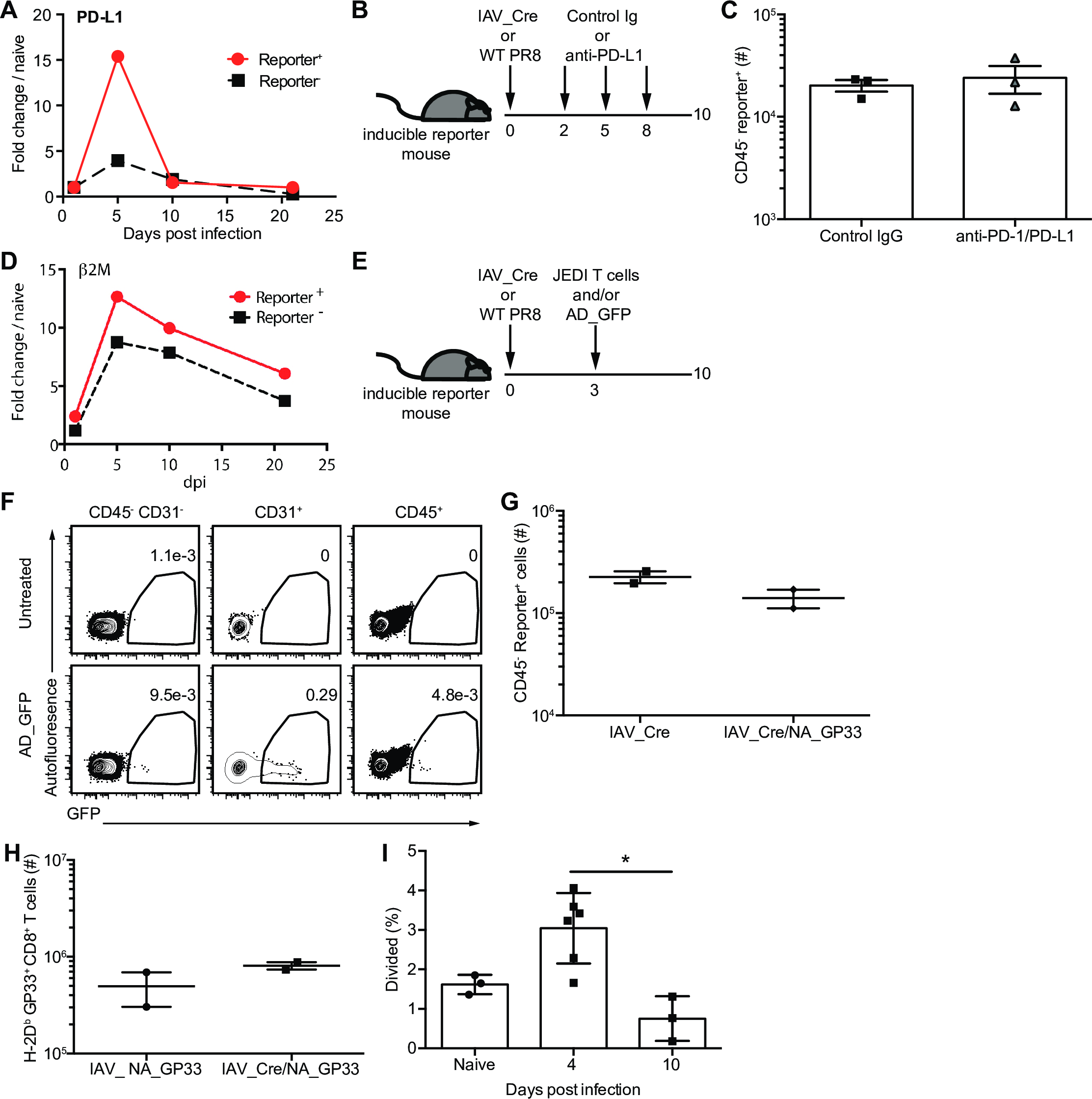

Supplement: S6 Fig — (A and C-D) Cre-inducible reporter mice were infected with IAV_Cre. (A and D) Live, CD45- reporter+ and reporter- cells were FACS sorted from IAV_Cre infected mice at indicated dpi and assessed by mRNA-seq.[2] (A) mRNA reads were analyzed for CD247 (PD-L1) at indicated dpi. (B) Schematic of control Ig or anti-PD-L1 treatment of infected mice in Fig 4. (C) On 2, 5 and 8 dpi mice were treated with PD-1 and PD-L1 blocking antibody or control IgG. Number of CD45- reporter+ cells at 10 dpi. (D) mRNA reads were analyzed for b2m (β2M) at indicated dpi. (E) Schematic of JEDI T cell transfer and AD_eGFP infection in Fig 5. (F) Representative flow cytometry plots of lung CD45- CD31-, CD31+ and CD45+ cells 7 days after AD_eGFP vaccination. (G-I) Cre-inducible reporter mice were infected with IAV_Cre, IAV_Cre/NA_GP33 or IAV_NA_GP33. (G) Number of lung CD45- reporter+ cells at 4 dpi. (H) Number of lung CD8+ CD44+ H-2Db-GP33+ T cells at 10 dpi. (I) CD45- CD31- Reporter+ cells were sorted from lungs of mice infected 4 or 10 days prior with IAV_Cre/NA_GP33. Sorted cells were mixed with in vitro activated P14 CD8+ T cells labeled with CTV. After 48 hours cells were analyzed by flow cytometry. Percentage of P14 CD8+ T cells that have divided. Data (A and D) were obtained from ref [2]. The results (C) are from 1 experiment with 3 mice per group (± SEM). The results (G) are representative of 3 independent experiments (± SEM). The results (H) are from 1 experiment with 2 mice per group (± SEM). The results (I) are representative of 2 independent experiments (± SEM). (TIF) [file ppat.1008077.s006.tif]
